# Traumatic Brain Injury: Mechanisms of Glial Response

**DOI:** 10.3389/fphys.2021.740939

**Published:** 2021-10-22

**Authors:** Rodrigo G. Mira, Matías Lira, Waldo Cerpa

**Affiliations:** ^1^Laboratorio de Función y Patología Neuronal, Departamento de Biología Celular y Molecular, Facultad de Ciencias Biológicas, Pontificia Universidad Católica de Chile, Santiago, Chile; ^2^Centro de Excelencia en Biomedicina de Magallanes (CEBIMA), Universidad de Magallanes, Punta Arenas, Chile

**Keywords:** traumatic brain injury, glia, astrocytes, microglia, oligodendrocytes

## Abstract

Traumatic brain injury (TBI) is a heterogeneous disorder that involves brain damage due to external forces. TBI is the main factor of death and morbidity in young males with a high incidence worldwide. TBI causes central nervous system (CNS) damage under a variety of mechanisms, including synaptic dysfunction, protein aggregation, mitochondrial dysfunction, oxidative stress, and neuroinflammation. Glial cells comprise most cells in CNS, which are mediators in the brain’s response to TBI. In the CNS are present astrocytes, microglia, oligodendrocytes, and polydendrocytes (NG2 cells). Astrocytes play critical roles in brain’s ion and water homeostasis, energy metabolism, blood-brain barrier, and immune response. In response to TBI, astrocytes change their morphology and protein expression. Microglia are the primary immune cells in the CNS with phagocytic activity. After TBI, microglia also change their morphology and release both pro and anti-inflammatory mediators. Oligodendrocytes are the myelin producers of the CNS, promoting axonal support. TBI causes oligodendrocyte apoptosis, demyelination, and axonal transport disruption. There are also various interactions between these glial cells and neurons in response to TBI that contribute to the pathophysiology of TBI. In this review, we summarize several glial hallmarks relevant for understanding the brain injury and neuronal damage under TBI conditions.

## Introduction

Traumatic brain injury (TBI) is the brain damage or alteration in normal brain function due to external forces such as the action of direct hits, acceleration or deceleration, or penetrating objects (Blennow et al., 2016). The world incidence of TBI is around 10 million cases each year and it is constantly increasing (Hyder et al., 2007); for example, in the United States, a total of 2.53 million cases of TBI were reported in 2014 (Capizzi et al., 2020). Among the leading causes of TBI, we can find vehicle accidents, falls, episodes of violence, and contact sports, affecting mainly people older than 75 years old, children, and young males (Maas et al., 2008; Coronado et al., 2015; Capizzi et al., 2020).

Traumatic brain injury could be classified as mild, moderate, and severe depending on clinical criteria such as state of consciousness, amnesia, and other neurological symptoms (Blennow et al., 2016). Mild traumatic brain injury (mTBI) is the most common form of TBI, encompassing 80–90% of all cases. mTBI cause an acute disruption of brain function, with or without a brief loss of consciousness, confusion, and symptoms that could persist for up to 1 year after the event (Silverberg et al., 2019). In addition, a minority of patients develop the post-concussion syndrome, which includes long-term organic and psychogenic symptoms including cognitive and behavioral changes. Nowadays, it is widely accepted that repeated mTBI may have long-term consequences such as the development of neurodegenerative diseases such as Parkinson’s Disease, Alzheimer’s Disease (AD), Chronic Traumatic Encephalopathy (CTE), among others (Kamins and Giza, 2016).

In the central nervous system (CNS), neurons are not the only cell type, and importantly, more than half of brain volume are glial cells (Jessen, 2004). In the CNS, we can find several types of glial cells: astrocytes, microglia, oligodendrocytes, and Neuron Glia-antigen two positive cells (NG2 + or polydendrocytes, or oligodendrocyte progenitor cells) (Peters, 2004).

Astrocytes play many roles in CNS, where they are closely associated with pre and postsynaptic terminals, constituting the tripartite synapse. Astrocytes perform potassium and glutamate buffering, support neuron metabolism, and release soluble factors known as gliotransmitters that modulate the synapse. Finally, astrocytes establish a link between the extracellular brain milieu and peripheral blood in the blood-brain barrier (BBB) (Chung et al., 2015; Ben Haim and Rowitch, 2017; Vasile et al., 2017).

Microglia are the main immune component in the CNS, acting as a phagocytic mononuclear cell. Microglia express a set of immune receptors to recognize pathogens, cytokine, chemokines, and complement receptors to develop their function. These cells are highly dynamic, and once activated, they can suffer morphological changes along with changes in protein expression and secretion, releasing pro and anti-inflammatory mediators (Colonna and Butovsky, 2017).

Oligodendrocytes are the producers of myelin in the CNS, although other functions have been described, such as the metabolic support of axons. Myelination is responsible for the electrical isolation of axons leading to an enhancement in the conduction speed of nerve impulses (Simons and Nave, 2015). NG2 cells are progenitor cells for oligodendrocytes, but they can localize in brain areas where myelination is not required. Interestingly, these cells express voltage-gated channels to generate and propagate action potentials; although this issue is still debated (Peters, 2004; Eugenin-von Bernhardi and Dimou, 2016; von Bernhardi et al., 2016).

Traumatic brain injury alters the function of the brain not only affecting the neuronal function. The important neuroinflammation observed in TBI as well as, the demyelination of white matters tracts highlights the role of glial cells to TBI. Here, we will describe the mechanisms involved in the response of different glial cells to TBI, their contribution to brain damage and the open questions to be explored.

## Traumatic Brain Injury Overview and Inflammation

Traumatic brain injury pathophysiology involves two types of damage: primary and secondary. Primary damage refers to the brain damage produced by the external force itself. It includes increases in intracranial pressure, hemorrhages, edema, and strains of the neural and vascular tissue leading to axonal injury [diffuse axonal injury (DAI)] (Nakagawa et al., 2011; Young et al., 2015). As a consequence of the strains of the axons, the axonal transport is blocked, leading to the disruption of organellar trafficking and direct intracellular damage (Meythaler et al., 2001).

On the other hand, secondary damage refers to cellular and molecular events that occur hours to days after TBI and includes further axonal degeneration and demyelination, neuroinflammation, oxidative stress, mitochondrial and synaptic damage, and protein aggregation. Importantly, secondary damage mechanisms are responsible for the persistence of symptoms and increase vulnerability to new brain trauma or other neurodegenerative disorders (Maas et al., 2008).

Regarding protein aggregation, TBI has been considered as a tauopathy due to the hyperphosphorylation, misfolding, and mislocalization of the protein tau, an axonal microtubule-associated protein (Luo et al., 2014; Mouzon et al., 2014; Josephs et al., 2017; Kovacs, 2017). Tau can form aggregates that are toxic to the cell and, importantly, can spread to other neurons, acting like a prion protein (Pavlova et al., 2016; Wang and Han, 2018). Also, amyloid-β (Aβ) aggregates, a product of the amyloidogenic processing of amyloid precursor protein (APP), have been associated with TBI (Abu Hamdeh et al., 2018; Shishido et al., 2019). The mechanism by which TBI induces the formation of protein deposits of tau and Aβ is still unknown but is strongly related to the development of CTE and AD (Grant et al., 2018).

Synaptic changes are also observed after TBI, such as decrease in the excitatory synaptic neurotransmission (Gao et al., 2011; Titus et al., 2016; Mira et al., 2020; Lira et al., 2021) in the hippocampus as well as impaired synaptic plasticity (Schwarzbach et al., 2006; Aungst et al., 2014). Along with synaptic deficit and protein deposition, oxidative damage and mitochondrial dysfunction are also characteristics of TBI. Brain cells showed protein and lipid modifications proper of oxidative damage by reactive oxygen and nitrogen species (Globus et al., 1995; Singh et al., 2006; Ohta et al., 2013), as well as decrease ATP production (Gilmer et al., 2009; Hubbard et al., 2019; Mira and Cerpa, 2020), leading to cell damage and apoptosis.

An important feature of TBI is the development of neuroinflammation, mainly due to glial activation. Neuroinflammation is defined as the innate immune response in the CNS to clear the system from damaged cells and infections. It is crucial for the regeneration of the CNS, but when neuroinflammation is chronic, it is detrimental to cells (Yang and Zhou, 2019). Early in TBI research, there has been evidence of neuroinflammation in the brain (Ott et al., 1994), such as proinflammatory cytokines production (Hans et al., 1999; Knoblach and Faden, 2000; Lloyd et al., 2008). Close-head injury induces the production of tumor necrosis factor α (TNF-α) and interleukin-6 (IL-6), two known proinflammatory cytokines (Shohami et al., 1994), the production of eicosanoids, proinflammatory mediators derived from phospholipids (Shohami et al., 1989), and inflammasome activation downstream innate immune receptors (Xu et al., 2018). After severe TBI, anti-inflammatory cytokine interleukin-10 (IL-10) was found in cerebrospinal fluid (CSF), as well as IL-6 and transforming growth factor β (TGF-β), suggesting a modulatory effect of neuroinflammation during TBI (Csuka et al., 1999). Time-course analysis of cytokines after controlled-cortical impact in mice showed that interleukin 1β (IL-1β) rise to peak levels within 6 h to 1 day after the injury, while TNF-α mRNA showed a steady increase until 3 days after injury. Anti-inflammatory cytokines mRNA insulin-like growth factor 1 (Igf-1) and lectin galactosidase-binding soluble 3 (Lgals3) showed a peak 3 days after the injury as well as the immunoregulatory interleukin 4 receptor alpha (Il4ra) (Taib et al., 2017), highlighting the differences in temporal response and importantly, that after an acute release of proinflammatory mediators, the immunoregulators and anti-inflammatory proteins take place. Moreover, treatment of animals submitted to TBI with IL-10 improves neurological outcome and reduces proinflammatory cytokine IL-1β (Knoblach and Faden, 1998), suggesting that neuroinflammation is in part responsible for neurological damage.

An important feature of neuroinflammation is the reactivity of two types of glial cells, astrocytes and microglia. The immunoreactivity of the astrocytic marker glial acidic fibrillary protein (GFAP) (Luo et al., 2014) and the microglial marker ionized calcium-binding adaptor molecule 1 (Iba-1) (Aungst et al., 2014; Chen et al., 2014; Mouzon et al., 2014; Broussard et al., 2018) increase in response to TBI In addition, periphery immune cells migrate to the site of damage such as neutrophils (Clark et al., 1994), inflammatory leukocytes (Soares et al., 1995; Schwarzmaier et al., 2013), macrophages, dendritic cells, and T cells (Jin et al., 2012) that contribute in brain damage (Chou et al., 2018; Kramer et al., 2019). Another important component of the immune system is the complement system and membrane attack complex; the Tomlinson group showed that these mediators are responsible for chronic inflammation propagation, proposing this pathway as a possible therapeutic target (Alawieh et al., 2018).

Glial cells play a key role in the regulation of synaptic transmission, myelination, and axonal health, and importantly in neuroinflammation, highlighting the crucial study of these cells in the pathophysiology of TBI. A more detailed description of the deregulation of glial cells in the CNS is at the center of the next sections.

## Traumatic Brain Injury and the Astrocytic Response

**FIGURE 1:**
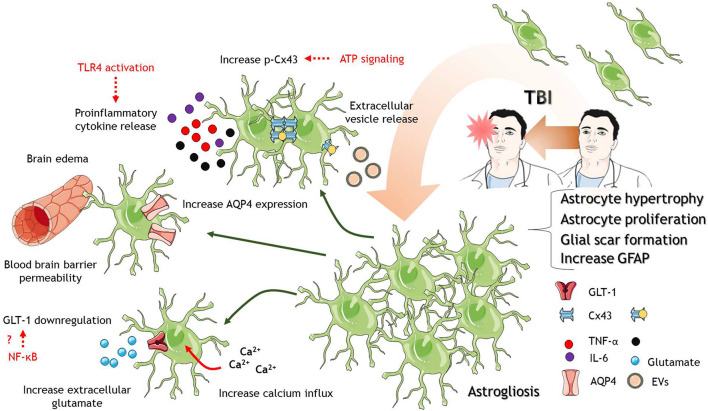
Astrocytes in response to TBI. After TBI of different severities, astrocytes are activated where they proliferate and increase in size. Astrocyte proliferation and activation lead to the formation of a protective glial scar. Several mechanisms at the molecular level accompanied astrocytic activation. Between them, increase calcium influx into astrocytes, downregulation of glutamate transporter GLT-1, the release of proinflammatory cytokines and TLR4 activation, increase p-Cx43 levels, increase AQP expression, and disruption of the blood-brain barrier that contributes to brain edema. The cartoon was made using SERVIER Medical Art templates smart.servier.com.

A key feature of TBI is the increase in GFAP immunostaining, indicating activation and/or proliferation of astrocytes. This process is known as astrogliosis as it has been widely documented after TBI of different severities (Chen et al., 2014; Luo et al., 2014; Mouzon et al., 2014; Carron et al., 2016; Broussard et al., 2018; Figure 1). It has also been described that astrocytes in the lesion site could derive not only from old pre-existing astrocytes but also from polydendrocytes, indicating astrocyte generation from progenitor cells early after TBI (Kim et al., 2012). The proliferation of astrocytes is possibly due to Notch signaling pathway action, including endothelin B, the transcription factor STAT3, and the Wnt signaling regulator Dixdc1 (Rogers et al., 2003; LeComte et al., 2015; Levine et al., 2016; Lu et al., 2017).

Astrocytes not only proliferate in response to TBI but also increase their size. The hypertrophy in astrocytes has been related to aberrant neurogenesis in the hippocampus, including ectopic growth and migration of newborn neurons after TBI (Robinson et al., 2016). Other studies have also indicated that atypical astrocytes are responsible for spontaneous seizure and epileptiform activity after TBI (Shandra et al., 2019), suggesting that altered astrocytic number and protein profile impact in neuronal function leading to long-term defects with phenotypic sequelae. But what is the role of astrocyte proliferation and activation after TBI? It has been widely accepted that the formation of an astroglial scar is a protective mechanism to avoid spreading of secondary damage to other brain regions (Faulkner et al., 2004). The formation of the scar gives important inflammatory mediators to remove damaged tissue and gives way to regeneration (Sofroniew, 2005; Myer et al., 2006). However, they could also exacerbate negative outcomes, for example, contributing to excitotoxicity, the spread of damage to distal sites of the lesion, neuroinflammation, and edema.

Among the damaging roles of astrocytes in TBI pathophysiology is their contribution to excitotoxicity. After TBI, there is an increase in the extracellular concentration of excitatory amino acids (Bullock et al., 1998; Amorini et al., 2017), which could be partially explained by the release of excitatory aminoacids from astrocytes or impaired glutamate buffering of astrocytes (Yi and Hazell, 2006). In the brain, there are five glutamate transporters or excitatory amino acids transporters (EAAT), and astrocytes express two of them, EAAT1 (GLAST) and EAAT2 (GLT-1) (Danbolt et al., 2016). Studies in postmortem human brain tissue derived from TBI patients have demonstrated decreased expression of EAAT1 and 2 in astrocytes and microglia (Ikematsu et al., 2002; van Landeghem et al., 2006; Beschorner et al., 2007), which could contribute to impaired glutamate buffering. Mouse models of TBI have corroborated these observations (Rao et al., 1998; Li et al., 2015), and it could be reverted by treatment with ceftriaxone, an antibiotic that upregulates GLT-1 (Goodrich et al., 2013). In this regard, the increase expression in GLT-1 by ceftriaxone reduce post-traumatic seizures and gliosis (Goodrich et al., 2013). The mechanism by which ceftriaxone induce the expression of GLT-1 is still unknown, but interestingly, an enhance glutamate buffering reduce the activation of astrocytes as it is shown by decrease GFAP expression. The reduction in GLT-1 levels after TBI is regulated by the transcription factors nuclear factor-kB (NF-kB) and *N*-myc in adult and aged mice (Gupta and Prasad, 2016). The reduction in GLT-1 expression is accompanied by reduced expression in the potassium channel Kir4.1, indicating that both potassium and glutamate uptake by astrocytes are impaired after TBI (Gupta and Prasad, 2013; Figure 1). The transcription factor NF-kB is a known immune regulator in different cell types (Mitchell et al., 2016). It is interesting that the reduction in GLT-1 expression and the consequent increase in extracellular glutamate could be an effect of the neuroinflammation. In fact, the treatment of animals with compounds with anti-inflammatory properties increase the expression of GLT-1 in other neuropathologies including addiction (Arezoomandan et al., 2019) and multiple sclerosis (Gentile et al., 2018; Colombo et al., 2020). Thus, the inflammation evoked by brain trauma could promote the excitotoxicity and neuronal damage by down-regulating GLT-1 expression in astrocytes. In the same line, the knockdown of GLT-1 exacerbates neuronal death after TBI (Rao et al., 2001), but also it has been shown that glutamate uptake could be reduced without changes in GLT-1 expression (Dorsett et al., 2017). While the usage of several models to induce brain trauma could explain this discrepancy, there is also the possibility that not only the expression of GLT-1 is a key player in excitotoxicity but also the post-translational modifications by the action of kinases such as PKC and Akt (Dorsett et al., 2017), which need to be further explored.

On the other hand, astrocytes could contribute to the spreading of secondary damage to distal regions from the lesion. An *in vitro* model of TBI showed that distal astrocytes increase the activity of connexin-43 (Cx43) channels (Rovegno et al., 2015). These proteins assemble into hexameric channels that communicate the cytoplasm of one cell with another one and are an important communication pathway between astrocytes. However, connexins can also assemble into hemichannels that communicate the intracellular and extracellular space (Scemes and Spray, 2012). Interestingly, the increase activity of Cx43 channels was absent under inhibition of P2 purinergic receptors (Rovegno et al., 2015), suggesting the regulation of spreading damage by ATP signaling. In animal models, the inhibition of Cx43 using antisense oligodeoxynucleotides or siRNA reduces astrogliosis, edema and improves cognitive performance (Wu et al., 2013; Ichkova et al., 2019). Endogenously, the micro-RNA cluster miR-302 has been associated with the reduction of extracellular regulated kinase (ERK) phosphorylation to their substrates, including Cx43. The increase of miR-302 cluster improves TBI outcome, reducing ERK phosphorylation and levels of phospho-Cx43 (Chen et al., 2019), adding a new regulatory level to these channels. The phosphorylation of Cx43 by ERK increases the release of exosomes from astrocytes after TBI, which has shown neuroprotective effects (Chen et al., 2018, 2020; Figure 1). The key mediator inside exosomes could be a truncated version of Cx43 called GJA1-20k. This protein enhances astrocytic mitochondrial performance (Chen et al., 2020), and surprisingly, increase the transfer of mitochondria from astrocytes to neurons in an *in vitro* model of TBI (Ren et al., 2021). Using *in vivo* TBI animal models, the overexpression of GJA1-20k in astrocytes showed reduced neuronal death and improved cognitive performance (Ren et al., 2020). It is unclear if the transfer of mitochondria from astrocytes to neurons is a key player in the role of GJA1-20k *in vivo* after TBI, although it has been already demonstrated in stroke (Hayakawa et al., 2016). The precise mechanism of production of this truncated form of Cx43 during TBI is still poorly understood and remains as an open question in the field. It is widely accepted the role of Cx43 in the spread of secondary damage in ischemia and other diseases, and now in TBI become a considerable therapeutic target.

What are the signals that trigger astrocytic activation? A known signal that modulates astrocytic function is the ion calcium (von Bernhardi et al., 2016). Calcium enters astrocytes downstream the activation of metabotropic glutamate receptors and calcium release from internal stores. These calcium signals lead to the activation of the astrocyte and propagate this signal to other astrocytes (von Bernhardi et al., 2016). The decrease in GLT-1 activity and increase of extracellular glutamate could mediate an overactivation of metabotropic glutamate receptors and calcium influx. *In vitro* TBI model has shown that scratch trauma increases calcium influx and activates the signaling pathway c-Jun N terminal kinase (JNK)/c-Jun/AP-1 to upregulate GFAP expression and astrocyte activation (Gao et al., 2013). It has also been indicated that mechanical shear stress itself promotes the calcium influx through mechanosensitive calcium channels (Maneshi et al., 2015) and short transient receptor channel 3 (TRPC3) (Belkacemi et al., 2017). Calcium signals activate calcineurin phosphatase, which induces the activity of the Nuclear Factor of Activated T cells (NFAT). The activation of astrocytic NFAT seems to be responsible for synaptic damage after TBI, but apparently, it does not affect astrocytic activation (Furman et al., 2016), indicating that synaptic failure and astrocytic activation could involve two different pathways, and therefore, the overall mechanism of astrocytic response is still not entirely understood.

Regarding the immune functions of astrocytes, they express the innate immune receptor Toll-like receptor 4 (TLR4), and it is activated after TBI. The downregulation of TLR4 reduces the production of proinflammatory cytokines IL-6, IL-1β, and TNF-α, reducing brain edema and neuronal death. Importantly, knockdown of TLR4 reduces astrocyte proliferation and hypertrophy, reducing astrocyte activation (Jiang et al., 2018). Astrocyte swelling is a contributor to brain edema. Aquaporin-4 (AQP4) is mislocalized after TBI, changing from the perivascular domain (endfeet) to a more distributed expression in the processes and cell body. This mislocalization and swelling are avoided with an AQP4 inhibitor, acetazolamide (Glober et al., 2019). Other pathways that contribute to edema are the activation of NF-κB, which mediate swelling through the upregulation of the Na/K/2Cl cotransporter (NKCC) (Jayakumar et al., 2014), and the Transient receptor potential melastatin 4 (TRPM4), which is activated by cytosolic calcium and doubled astrocytic soma size (Gorse et al., 2018). Thus, astrocytes contribute to brain edema directly through several signaling pathways, including neuroinflammation. This is especially interesting, given that the modulation of TLR4 in TBI animal models have not covered yet the potential role in excitotoxicity. If the activation of TLR4 after TBI induces the translocation of NF-κB to the nucleus, not only overexpresses genes that mediate swelling and astrocytic activation, but it could also downregulate the expression of GLT-1 contributing to excitotoxicity and calcium-induced damage in a vicious cycle of damage triggered by neuroinflammation.

Micro-RNAs are also part of the development of TBI pathophysiology, but the knowledge of it, is rather low. miR-195 excels its activity in TBI by suppressing the anti-inflammatory Nod-like receptor (NLR) X1 function (Theus et al., 2017; He et al., 2021), but astrocytes can modulate miR-195 activity by transferring through extracellular vesicles a long-non-coding RNA, which in turn upregulates NLRX1 and reduces detrimental effects to neurons (He et al., 2021). Additionally, Korotkov et al. (2020) suggested that activated astrocytes promotes brain inflammation through miR142 and miR155 specifically in the secondary injury evoked after TBI induction. This is not surprising since astrocytes alongside microglia have been found promoting neuroinflammation for several decades by now. Finally, downregulation of miR-9-5p promote the proliferation of astrocytes and evoke the release of astrocyte-derived neurotrophic factors that promotes overall brain cell survival (Wu et al., 2021). The astrocytic proliferation during TBI also contributes to monocyte invasion due to alterations in the vasculature and contributing to immune response (Frik et al., 2018). However, astrocytes also can protect the BBB after TBI. Astrocytes upregulate the expression of fatty acid-binding protein 7 (FABP7) with a concomitant increase in caveolin-1 in endothelial cells. Both processes ameliorate neurological deficit, brain edema, and BBB permeability (Rui et al., 2019).

Several studies have related astrocytes with damage and recovery after TBI. Astrocyte activations contribute to neuroinflammation, edema, and both disruption and recovery of the BBB. They contribute to the spreading of secondary damage and decrease glutamate buffering, contributing to excitotoxicity. To date, several groups have suggested strategies to modulate astrocytic function/dysfunction in TBI animal models but still is debated the temporal response to astrocytes and their harmful or protective effects in neuronal survival and correct synaptic transmission.

## Traumatic Brain Injury and Microglial Activation

**FIGURE 2:**
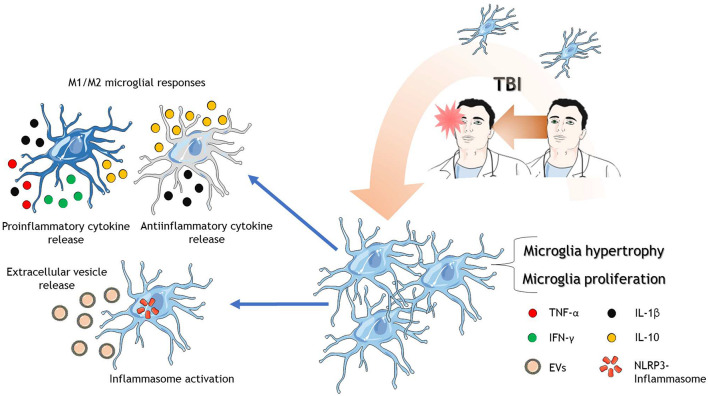
Microglia in response to TBI. After TBI of different severities, microglia are activated where they proliferate, increase in size and complexity. There are reports that microglia polarize into possible phenotypes, M1 and M2. M1 microglia release mainly proinflammatory cytokines and the anti-inflammatory cytokine IL-10. On the other hand, M2 microglia release mainly anti-inflammatory cytokines and the proinflammatory cytokine IL-1β. Microglia also activate the NLRP3-inflammasome protein complex and release extracellular vesicles to spread neuroinflammation. The cartoon was made using SERVIER Medical Art templates smart.servier.com.

Microglial cells can remove cell debris and toxic substances, helping to maintain correct brain homeostasis. TBI is recognized to deliver cellular damage, and these cells, in turn, release Danger-associated molecular patterns (DAMPs), which can become an outstanding inflammatory stimulus, resulting in further tissue damage (Solito and Sastre, 2012). When an injury occurs, microglial cells activate rapidly, modifying their normal morphology into a ramified cellular structure and larger cell body (Xu et al., 2017; Figure 2). Microglia are the primary mediators of the brain’s innate immune response and react to TBI within minutes. In fact, microglia might be recognized as the first-line defense since the fast and local response is observed upon TBI induction (Davalos et al., 2005). In this injury context, microglia cells can proliferate and migrate into the injury location. Also, microglia become polarized and induce the release of cytokines depending on the activation state (Kim et al., 2015). The fast response of these cells can be observed chronically by weeks or months after brain injury (Bodnar et al., 2018; Henry et al., 2020; Ritzel et al., 2020) depending on TBI severity.

Up until now, two polarization states of activated microglia have been described, depending on the stimuli they receive, the M1 and the M2 phenotypes. The M1 phenotype secretes a significant amount of proinflammatory cytokines [interferon-γ (IFN-γ), TNF-α, and IL-1β] and chemokines (Xu et al., 2017) but barely secrete the anti-inflammatory cytokine IL-10 (Chio et al., 2015). On the other hand, the M2 phenotype is more complex ranging from anti-inflammatory response to mixed anti and proinflammatory responses (Bell-Temin et al., 2015). Altogether, the M2 phenotype appears to be neuroprotective by an anti-inflammatory effect (Loane and Kumar, 2016).

Interestingly, redirection of microglia polarization to the M2 phenotype seems to be a good strategy to improve outcomes in TBI animal models. In this regard, the peroxisome proliferator-activated receptor γ (PPAR-γ) pathway has been associated with the attenuation of inflammation by promoting M2 polarization of microglia after TBI (Wen et al., 2018). Despite the neuroprotective contribution of M2 microglia, if the insult cannot be controlled, M2 microglia became “overwhelmed,” and chronic proinflammatory M1 activity arises, and long-term damage takes place (Donat et al., 2017; Simon et al., 2017). These same groups have discussed some controversies within these results, noticing that temporal, single-cell patterning might be a challenge for the future. Microglia subtypes can change their phenotype very rapidly depending on the insult or the evolution of the injury, which determines the type of molecules that triggers these phenotypic changes. Therefore, it is unclear how microglia subtypes emerge and change in the development of the injury.

Gene-profiling studies strongly implicate early microglial activation after TBI. Markers of microglial activation (CD68, MHC-II) and chemokine expression (CXCL10, CXCL6), among others, have been shown to increase 3 h after TBI (Israelsson et al., 2008). Jassam et al. (2017) pointed out in 2017 that gene expression profiles have not been evaluated using techniques such as single-cell RNA sequencing. After that, few single-cell (Lee et al., 2019) experiments have been performed. By isolating cells using cell sorting, it is concluded that Cxcl1 and Il1b (among others) gene expression was increased in hippocampal microglia 24 h after TBI (Arneson et al., 2018). Single-cell temporal expression pattern of microglia inflammatory genes also demonstrates that a biphasic pattern of IFN-γ, interleukin-4 (IL-4), and IL-10 is observed between 14- and 60-days post-injury, suggesting that proinflammatory signals precede anti-inflammatory microglial functions (Izzy et al., 2019). In this study, they proposed that classic microglial activation (i.e., M1/M2 phenotype) might not represent what is occurring in TBI pathophysiology since M1 vs. M2 classification could be oversimplified in this context (Izzy et al., 2019).

Microglial inflammasome proteins are part of the microglia activation processes in TBI as well. The inflammasome is an intracellular multiprotein complex that mediates immune responses against DAMPs (Olcum et al., 2020). Inflammasome comprises a sensor protein: nucleotide-binding, Leucine-rich repeat-containing proteins (NLRP), an adaptor molecule: apoptosis-associated speck-like protein (ASC), and an effector enzyme (caspase-1) (Broz and Dixit, 2016). The inflammasome can be activated by several molecules, including DAMPs, potassium efflux, intracellular calcium increase, among others (Olcum et al., 2020), all of them being present in TBI pathophysiology (Carvajal et al., 2016; Relja and Land, 2020). After the activation of the inflammasome, common consequences include the secretion of cytokines and pyroptosis (Olcum et al., 2020). Interestingly, NLRP3 microglial inflammasome activation is observed as early as 24 h post-injury (Lee et al., 2018b; Mortezaee et al., 2018; Xu et al., 2018; Lee et al., 2019), which causes pyroptotic cell death in a severe TBI murine model (Lee et al., 2019). Using the same TBI model, NLRP3, ASC, and active caspase-1, increased expression was observed 24 h after injury in activated microglia with sustained microglial activation for 12 weeks (Lee et al., 2018b). NLRP3 inhibition was able to reduce inflammation and rescue neurons from microglia-mediated cell death (Mortezaee et al., 2018; Xu et al., 2018). It seems that inflammasome activation is a key event within TBI pathophysiology development, and therefore it should get more attention in the future.

Besides cytokine release *per se*, microglia can release extracellular vesicles (EVs) during inflammation (Yang et al., 2018; Figure 2). Indeed, microglial-derived microparticles -a type of EV- are part of the inflammation process in TBI (Kumar et al., 2017) by carrying proinflammatory cytokines, which in turn exacerbates the inflammation that the system already has. Also, the data indicate that these microparticles can propagate and rapidly disseminate brain inflammation due to the activation of non-activated microglia. Inflammasome proteins have also been detected in EVs obtained from the CSF of TBI patients (de Rivero Vaccari et al., 2016). Although we cannot conclude that these EVs are released by microglia, it is plausible to hypothesize that microglia could be releasing these inflammasome-containing EVs. On the other hand, microglial microparticles could also serve as a treatment for TBI. These microparticles, which contain miR-124—3p, can reduce inflammation and improve the neurologic outcome after TBI (Huang et al., 2018). Besides, several brain-produced EVs that carry anti-inflammatory molecules have been discovered and used to reduce inflammation in the TBI context (Panaro et al., 2020). Thus, one could speculate that EVs containing anti-inflammatory molecules could be a therapeutic strategy in TBI-related inflammation. Advances in molecular profiling using micro-RNAs have shed light into the mechanisms that are intended to regulate or to be regulated by microglia inside TBI pathophysiology. A key member of micro-RNA lethal-7 family (let-7c-5p), known to regulate cell proliferation and apoptosis, was found to decrease after severe TBI induction. On the other hand, overexpression of let-7c-5p led to the inhibition of neuroinflammation and microglia activation due to the enhancement of microglial M2 polarization (Lv et al., 2018). Neuron-derived exosomes highly enriched with miR-21-5p after TBI triggers M1 microglial polarization (Panaro et al., 2020). Those M1 microglia gather at the damage site where neurons with increased miR-21-5p are localized (Harrison et al., 2016), and therefore a suggested cyclic cumulative damage between neurons and microglia is due to the transfer of miR-21-5p through exosomes (Panaro et al., 2020). On the other hand, exosomes containing miR-124-3p are usually released by microglia and promotes anti-inflammatory signals through M2 microglial activity, which in TBI context, those exosomes improve neurologic outcome and inhibit neuroinflammation (Huang et al., 2018; Li et al., 2019), reinforcing the role of M2 type microglia function in the recovery of the trauma. Indeed, miR-124 have been found to promote M2 polarization by inhibiting TLR4 immunological pathway after the triggering of the brain damage (Yang et al., 2019).

## Traumatic Brain Injury, Myelin, and Oligodendrocytes

One of the most common outcomes of TBI in patients is white matter degeneration, even more evident than gray matter alterations. Morphological changes in white matter are explained due to DAI or widespread axonal damage. Damage areas includes the corpus callosum, fornices, midbrain, pons, cerebellum, among others in head injury postmortem patients and in animal models of TBI where myelin loss is also evident (Blumbergs et al., 1989; Anderson and Bigler, 1995; Bramlett and Dietrich, 2002).

One way to study the injury to axons is the immunostaining of amyloid precursor protein beta (βAPP) given the disruption of fast axonal transport (Gentleman et al., 1993). Patients of mTBI, in postmortem analysis, showed βAPP positive immunostaining in axons along with axonal degeneration in fornices, explaining, in part, memory impairment (Blumbergs et al., 1994). Swollen axons have shown co-staining not only with βAPP but also with Aβ and the enzymes beta-site amyloid precursor protein cleaving enzyme (BACE) and presenilin-1 (PS1), indicating that axonal transport interruption by head trauma leads to the accumulation of APP and processing enzymes (Chen et al., 2004).

Interestingly, the function of myelination is also impaired. In a mouse model of mTBI, the conduction velocity through myelinated axons is decreased in the corpus callosum, which recovers partially 2 weeks after TBI. The fast-conducting component of compound action potentials is also decreased after mTBI, while the slow-conducting component increase, possibly due to the loss of myelinated axons (Marion et al., 2018). Alterations in node and paranode structure also change after mTBI, showing asymmetry between paranodes and the formation of heminodes (Marion et al., 2018).

**FIGURE 3:**
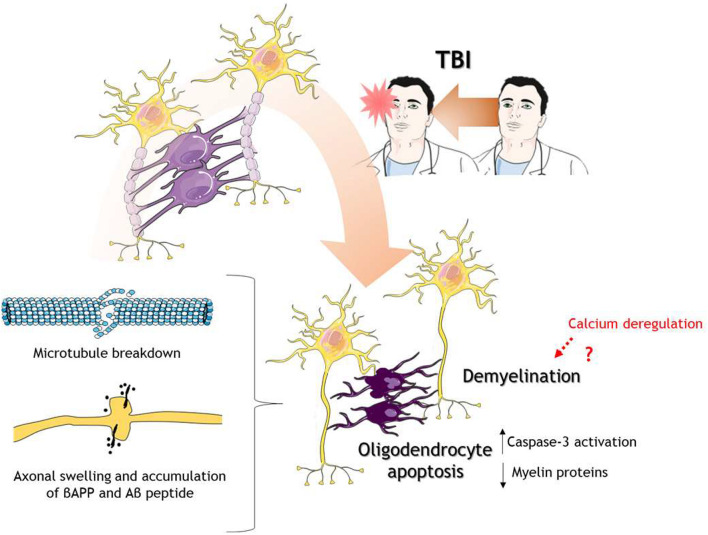
Oligodendrocytes in response to TBI. After TBI of different severities, oligodendrocytes go under the process of programmed cell death or apoptosis. Oligodendrocyte death caused decrease levels of myelin proteins leading to demyelination. There is also microtubule breakdown which translates into axonal transport disruption, axonal swelling, and accumulation of βAPP and Aβ peptides. The cartoon was made using SERVIER Medical Art templates smart.servier.com.

Due to oligodendrocytes are the myelin producers and the functional and structural support of axons in the CNS, alterations in these glial cells need to be considered (Figure 3). Studies in the last decade have shown that oligodendrocytes immunoreactivity using CC1 antibody, a mouse monoclonal antibody against adenomatous polyposis coli protein which label mature oligodendrocytes (Bin et al., 2016), is decreased after TBI in a rat model, and importantly, there was co-staining with caspase 3, indicating apoptosis of oligodendrocytes (Lotocki et al., 2011; Flygt et al., 2013; Figure 3). Classical white matter tracts such as corpus callosum, fimbria, and external capsule, showed decrease myelin levels using Luxol fast blue (LFB) staining and RIP antibody (Flygt et al., 2013), a mouse monoclonal antibody that recognizes 2′,3′-cyclic nucleotide 3′-phosphodiesterase used to label oligodendrocytes and myelin sheets (Watanabe et al., 2006). It has been shown that calcium is a regulator of myelin formation in the CNS, given that the retraction of myelin is mediated by calcium-induced calpain activation but also myelination is dependent on calcium transients (Baraban et al., 2018). Therefore, it is conceivable that calcium dysregulation in TBI could mediate in part the demyelination process.

On a temporal scale, the reduction in mature oligodendrocytes post-TBI is maintained for up to 2 weeks, but apoptosis of these cells seems to be more prolonged, extending up to 5 weeks after TBI (Dent et al., 2015). There is still no evidence that explains the sustained activation of apoptosis in oligodendrocytes. In the next section we will discuss some evidence regarding some mechanisms that explain the loss of oligodendrocytes, although their implications in sustained apoptosis are not clear. The loss of mature oligodendrocyte has also been reported in human brain tissue derived from patients with moderate to severe TBI (Flygt et al., 2016). In a mouse model of pediatric mTBI and repetitive mTBI, long-lasting defects in white matter tracts were found 60 days post-injury, including decrease length in the tracts and an increase in oligodendrocytes cells (Lee et al., 2018a).

The polydendrocytes have been identified as proliferative cells after brain trauma (Mierzwa et al., 2015). Olig2 is a transcription factor proper of oligodendrocyte lineage, including oligodendrocytes and polydendrocytes. Olig2 + cells proliferate 2 days after injury, reaching a peak 1 week after TBI and with long-lasting effects up to 3 months after injury (Dent et al., 2015). Other studies have indicated proliferation of polydendrocytes after 7 days post-injury (Flygt et al., 2013) remaining until 21 days post-injury (Flygt et al., 2017). Interestingly, polydendrocyte proliferation was also observed in human postmortem tissue from patients with moderate and severe TBI (Flygt et al., 2016). The precise molecular mechanism of NG2 cells proliferation and differentiation after TBI has not been explored yet. However, NG2 cells express ionotropic glutamate receptors α-amino-3-hydroxy5-methyl-4-isoxazole propionic acid (AMPA) and *N*-methyl-D-aspartate (NMDA) (Li et al., 2020a). The increase glutamate release observed in TBI could be a signal to modulate proliferation and/or differentiation of NG2 cells, either to oligodendrocytes, or other cell types such as neurons and astrocytes. It is well established that neural activity increases the proliferation of NG2 cells in juvenile and adult mice and promotes myelination (Gibson et al., 2014), but the contribution of decreased glutamatergic transmission and increase glutamate concentrations in a TBI scenario have not been explored. Two interesting treatments that have been shown to reduce oligodendrocyte death and recovery of myelin proteins are a neutralizing antibody of IL-1β (Flygt et al., 2018) and phospholipid supplementation (polyunsaturated fatty acids, docosahexaenoic acid, and eicosapentaenoic acid) in diet (Thau-Zuchman et al., 2019; Figure 3). These studies revealed the role of neuroinflammation in oligodendrocyte cell death, as well as phospholipid metabolism, to avoid the loss of myelin or enhance remyelination. The effect of neuroinflammation in myelin loss will be considered in the next section.

## Glial Interactions in Traumatic Brain Injury

Interestingly, glial responses to TBI are not isolated responses but probably integrated and coordinated. However, this issue has been barely studied, and it is a wide field of research to explore. The most studied crosstalk between glial responses is the astrocytic and microglial responses. Indeed, the rapid microglial response after TBI is dependent on extracellular ATP concentrations. The chemotactic response induced by ATP could be avoided with antagonists of astrocytic purinergic receptors or connexin inhibitors. The action of purinergic receptors on astrocytes is the release of more ATP and the spreading of calcium waves that lead to microglial recruitment, establishing control, and crosstalk between the responses of both cell types (Davalos et al., 2005).

Furthermore, homeostatic response to ATP levels has been indicated. Microglia could promote the downregulation of purinergic receptors in astrocytes, specifically P2Y receptors, decreasing ATP-induced signaling in astrocytes. The net effect is the transformation of astrocytes to a neuroprotective phenotype in favor of glial scar formation isolating damaged tissue (Shinozaki et al., 2017).

The initial microglial activation in response to TBI promotes astrogliosis and persistent inflammation (Witcher et al., 2018). Depending on the severity and the mechanism to generate the lesion, it is still unclear whether microglia promote astrocyte reactivity or downregulate astrocyte reactivity and cytokine release. To date, the crosstalk between both glial types is not resolved at the timing levels and further less at the mechanistic level, where purinergic receptors seem to be key mediators.

**FIGURE 4:**
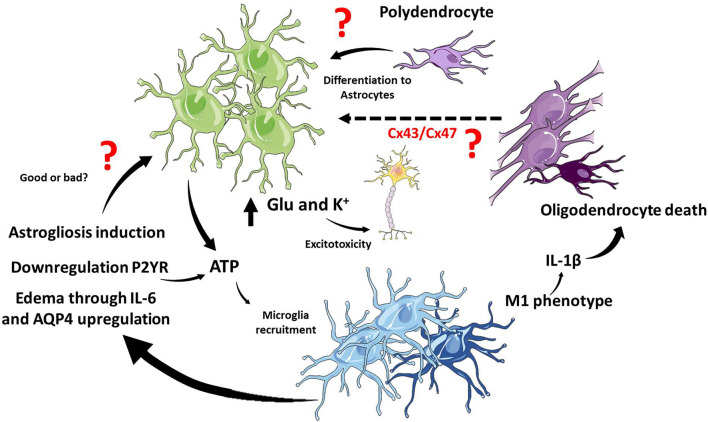
Glial interaction in TBI. In the TBI context, microglia and astrocytes interact through several pathways, and it is still not clear if these interactions potentiate or cushion tissue damage. ATP release from astrocytes induces microglial recruitment, and activated microglia induce astrogliosis and edema. On the other hand, microglia have been shown to regulate ATP release from astrocytes. M1 microglia phenotype and the release of IL-1β in white matter promote oligodendrocyte cell death. There is still unknown about the interactions between mature oligodendrocytes and astrocytes but connexins might play a role. Polydendrocytes, on the other hand, could proliferate and differentiate into astrocytes at the site of the lesion. The cartoon was made using SERVIER Medical Art templates smart.servier.com.

Astrocytes and Microglia communicate with each other through cytokines they release as well as other extracellular mediators. Two good examples will be described here. After TBI, neurons release high mobility group box protein-1 (HMGB1), a secreted chromatin protein released by immune cells and activates TLR4 in microglia leading to the release of IL-6. IL-6, in turn, activate astrocyte and increase the expression of AQP4, producing astrocyte swelling and contributing to brain edema (Laird et al., 2014; Figure 4). Other extracellular mediators are the EVs which are released from microglia and contribute to inflammation, but astrocytes also produce EVs. Recently, it has been described that exosomes derived from human astrocytes in response to TBI, containing miRNAs, inhibit neuroinflammation. Exosomes enriched in miR-873a-5p modulate microglial response and promote M2 phenotype leading to decrease neuroinflammation and a protective function through the inhibition of the NF-κB signaling pathway (Long et al., 2020).

Little is known about the crosstalk between oligodendrocytes and other glial cells in response to TBI. There are no studies that directly relate oligodendrocytes with astrocytes, and new insights have appeared between oligodendrocytes with microglia.

The activation of microglia and macrophages have been directly related to white matter injury (Johnson et al., 2013). Indeed, the inhibition of the microglial activation reduces white matter injury. In particular, the preservation of myelin is associated with a polarized M2 microglia phenotype (Wang et al., 2015). Recent evidence suggests that white matter and oligodendrocyte loss are dependent of M1 microglia. Upstream M1 polarization, the endoplasmic reticulum stress sensor protein kinase R-like endoplasmic reticulum kinase (PERK) in neurons, increases the release of interferon-β (IFN-β), which, in turn, induces M1 microglia activation and T-cell infiltration leading to white matter, myelin, and oligodendrocyte loss (Sen et al., 2020). We have previously exposed that the inhibition of IL-1β reduces oligodendrocyte loss, which is consistent with oligodendrocyte loss favored by proinflammatory environment and microglia M1 phenotype.

Despite the lack of evidence of a direct conversation between astrocytes and mature oligodendrocytes in the context of TBI, it has been widely documented that both cell types communicate with each other through connexins (Basu and Sarma, 2018; Fasciani et al., 2018; Li et al., 2020b). The astrocytic Cx43 join with their oligodendrocytic partner Cx47 in the formation of gap junctions in the CNS (Fasciani et al., 2018). Importantly, this interaction regulates both myelination and demyelination in health and disease (Basu and Sarma, 2018). Interestingly, the remyelination process improve in a mouse with the conditional deletion of astrocytic Cx43 in a context of demyelination induced by lysolecithin (Li et al., 2020b). As we previously mentioned in section “Traumatic Brain Injury and the Astrocytic Response,” after TBI increase the activity of Cx43 hemichannels, and the inhibition of Cx43 improve outcome in animal models. Thus, in the context TBI we could propose that Cx43 is a key molecular player in demyelination and probably oligodendrocyte apoptosis, which remain to be determined experimentally.

On the other hand, polydendrocytes or oligodendrocyte precursor cells also could activate in response to TBI. It is known and previously discussed that polydendrocytes could proliferate and differentiate to astrocytes (Kim et al., 2012). Interestingly, when gray matter and white matter suffered damage upon TBI, microglia proliferate preferentially in white matter, while astrocytes preferentially proliferate in gray matter. The lesion of white matter influences the proliferation of astrocytes in gray matter, and importantly, decreases polydendrocytes, indicating that astrocytes could be derived from these precursor cells (Mattugini et al., 2018). The process of differentiation of NG2 cells to astrocytes is different depending on the type of damage. Hackett et al. (2018) showed that after spinal cord injury a 25% of astrocytes derived from NG2 cells while this percentage decrease to 9% when in a model of autoimmune encephalomyelitis. In TBI the contribution of NG2 cells to astrocytes and importantly reactive astrocytes has been unexplored. Furthermore, what are the molecular players which take place to differentiate NG2 cells to astrocytes in this scenario is also an open question.

## Concluding Remarks

Traumatic brain injury is a critical health problem worldwide leading to death, and a risk factor in the development of neurodegenerative diseases. Due to the heterogeneity in TBI cases the generation of a model that faithfully replicates the pathophysiology has been a complex issue. Moreover, TBI interfere with the function of the brain itself, not only neurons. Most of the reports so far published have focused on the response of specific cell types, but few reports have intended to evaluate the interplay between these cells in TBI pathophysiology. Here, we had reviewed individual cellular and molecular responses of glial cells and ultimately present evidence of their interactions in the TBI context.

Upon TBI, astrocytes and microglia are activated from short to long time span depending on the severity of the injury. In the brain, a proinflammatory environment develops along with the astrocytic and microglial proliferation. The neuroinflammation spread across the brain and impact in the potassium and glutamate homeostasis, as well as, promoting demyelination and oligodendrocyte loss. The glial responses to TBI are prominent mediators of neuronal death and survival balance, and the close relationships between them are poorly explored. There is a wide research area to cover, combining spatial and temporal aspects of cellular response after TBI.

Another factor that adds complexity to this topic is the severity of TBI and the model used, which also showed different spatial and temporal effects on different cell types.

Prospective research is underway to understand better what are the key cellular processes that could be potentially pharmacological targets to treat TBI patients. There is a wide variety of possible molecular targets to explore including miRNA and EVs. The aim will be to offer TBI patients a better recovery from head trauma and fewer cumulative effects with age.

## Author Contributions

RM and WC conceived the idea. RM and ML performed the literature review and wrote the manuscript. WC supervised, reviewed, and edited the manuscript. All authors contributed to the article and approved the submitted version.

## Conflict of Interest

The authors declare that the research was conducted in the absence of any commercial or financial relationships that could be construed as a potential conflict of interest.

## Publisher’s Note

All claims expressed in this article are solely those of the authors and do not necessarily represent those of their affiliated organizations, or those of the publisher, the editors and the reviewers. Any product that may be evaluated in this article, or claim that may be made by its manufacturer, is not guaranteed or endorsed by the publisher.
